# Person-Centered Care From a Relational Ethics Perspective for the Delivery of High Quality and Safe Healthcare: A Scoping Review

**DOI:** 10.3389/fpubh.2020.00044

**Published:** 2020-03-06

**Authors:** Gianpaolo Tomaselli, Sandra C. Buttigieg, Aldo Rosano, Maria Cassar, George Grima

**Affiliations:** ^1^Department of Health Services Management, Faculty of Health Sciences, University of Malta, Msida, Malta; ^2^Italian National Agency for Regional Health Services (AGENAS), Rome, Italy; ^3^Department of Nursing, Faculty of Health Sciences, University of Malta, Msida, Malta; ^4^Faculty of Theology, University of Malta, Msida, Malta

**Keywords:** person-centered care (PCC), patient-centered care, ethics, relational ethics, patient safety, quality of care, health systems

## Abstract

**Background:** The aim of this scoping review is to explore whether or not person-centered care (PCC), in its quest to deliver high quality and safe health care, has a relational-ethics perspective. To do so, we first need to relate the extant literature pertaining to PCC and relational ethics. To this extent, the specific features that define PCC and relational ethics were identified. PCC dimensions include: patient and provider concordance, improved health outcomes, improved patient safety, individual expectations, patients' integration within the environment, patient as a person, patient as an active part of society, dialogue and interaction, sharing experience, and documentation of patient's (person's) narrative. Relational ethics framework includes the following actions: mutual respect, engagement, embodied knowledge, environment, and uncertainty.

**Methods:** Data were retrieved through multiple keywords search on PubMed, Medline, and Scopus. Inclusion/exclusion criteria were set, and these were based on year of publication (2008–2018), language, paper focus, research method and document types. A total of 23 articles (*N* = 23) were selected and reviewed. Content analysis was conducted in order to identify and compare the main features of PCC and relational ethics.

**Results:** The most important relational ethics action referred to in conjunction with PCC features is environment (referred to as person's integration within a social environment/community). This is followed by mutual respect, engagement and embodied knowledge. These were the salient relational ethics actions both directly and indirectly linked to PCC. Uncertainty was the less recurrent relational ethical action mentioned.

**Conclusions:** This paper revealed that while PCC features embrace most of the relational ethics approaches, these are not exploited in their entirety and therefore PCC emerges as a unique ethical stance in healthcare. PCC's ethical approach goes beyond what is explained within provider-patient relational ethics and emphasizes that the patient is an active person and a partner in care with capabilities and resources. This distinction enables us to explain the paradigm shift from “patient-centered” to “person-centered” care. The healthcare provider partnership and co-creation of the healthcare plan contributes to the delivery of high quality, safe and cost-contained healthcare.

## Background

Person-centered care (PCC) is a responsive and respectful approach to care taking into consideration persons' demands, preferences, and principles (1). It contributes to patients' empowerment by involving them in decision-making processes on treatment plans (2–4). In this respect, PCC is a development of the original concept of patient-centered care—which is defined in literature as “understanding the patient as a unique human being” (5). Patient-centered care is the most well-known concept in literature, however this approach considers the patient as a more passive recipient of care and its focus is merely on the medical treatment and diagnosis. PCC goes beyond patient-centered care as it has an ethical foundation and sees the person (not just the patient) as an active part of medical treatment and considers his/her needs, family, history, strengths and weaknesses. According to McWhinney and Stewart, who explored PCC outcomes within health systems (6), PCC sees the patient at the center of medical care, as well as of education and research (2003).

But it was Ekman et al. (7), who took PCC to a different and higher ethical level by basing their philosophy on Amartya Sen's capability approach, namely that the person is considered as someone who has capacities or is capable (8–11). This approach finds its roots in Aristotle's principle of human flourishing where quality is not an act, but it is a habit and healthcare providers are called to improve their emotional intelligence so as to be able to take care of other persons' needs (12). Furthermore, Aristotle's perspective of care assumed that each person has to be considered individually and as a special case. Persons not only have needs but they are repositories of capabilities and resources that can be engaged, and this is the point of departure in PCC. Thus, Ekman et al.'s philosophy of PCC (7), sees the patient as a capable human being, even if he or she is very weak or sick. Moreover, the patient with the healthcare provider/s are seen as partners in care and co-creators of the healthcare plan. According to Ekman et al., in order to create this partnership, health providers have to listen to the patients by taking into consideration their experiences, conditions and also individual expectations (13). A mutual health plan is to be agreed upon and has to be updated continuously and documented. Thus, responsibilities are equally distributed between the patient and the provider/s (14, 15).

According to Ekman et al. (7), PCC is characterized by three key concepts, namely: (i) person-provider partnership; (ii) inclusion of patient's narrative; and (iii) documentation of patient's (person's) narrative. However, further components can be identified while describing PCC features. These include: patient and provider concordance; improved health outcomes; improved patient safety; individual expectations; patients' integration within the environment; patient as a person; patient as an active part of society; dialogue and interaction; sharing experience; documentation. Table 1 summarizes PCC features according to Ekman et al. (7) framework.

**Table 1:** Person-centered care features.

**Person-centered care concepts**	**Features**
Partnership	Patients and providers concordance Patients integration within the environment Patient as a person Patient as an active part of his/her healthcare and of society
Patient narrative	Individual expectations Dialogue and interaction Sharing experience
Documentation	Documentation Improved health outcomes Improved patient safety

In order to better understand PCC and implement this approach within healthcare organization, it needs to be considered from an ethical perspective, which lies in the healthcare principle of recognizing self-fragility and coherence in life (7, 16). Some authors sustain that PCC already embodies ethics within itself (17), considering that its foundations lie in the human relationship between patients and providers—which is based on the key consideration of patients as persons. Thus, the person is not considered individualistically but in relation with others and embodied, interdependent and connected with the social environment and context (18). This approach determines the paradigm shift from the biomedical model of care—which is characterized by the dominance of the physician—to the biopsychosocial model where the person takes a central role in the decision-making processes regarding medical treatments (19). In this regard, biochemical alterations are not the only determinants of illnesses, but they need to be considered together with psychosocial variables and the relationship between the patient and the professional (20) plays a key role in influencing medical outcomes (6, 21–23). According to this philosophy, a correct diagnosis is only partially dependent of the healthcare provider's clinical tasks, which need to be complemented with proper dialogue and interaction with patients (24–26) in order to provide a more effective diagnosis (27). Thus, the ethical component represents a key aspect of PCC. Ethics refers to the principle of doing the right thing, establishing the right kind of relationships and/or destroying what is wrong according to the basic concepts and fundamental principles of decent human conduct (28). Within the broad concept of ethics, the relational ethics theory assumes a key importance in the context of PCC. Building on Bergum and Dossetor (29) and Pollard (18), relational ethics refers to those relationships, which are considered better than others and aim at stimulating growth, healing, and health. Furthermore, according to Evans (30), relational ethics is defined as an action ethics that is placed within the interpersonal relationships. The action ethics includes engagement, mutual respect, embodiment, and interdependent environment. The leading principle is that ethical decisions and actions should be made in the context of relationships. According to this perspective, the focus of relational ethics is not the action itself but the relationship (31). Thus, the relational ethics framework includes the following actions: mutual respect; engagement; embodied knowledge; environment; and uncertainty [(18), p. 364]. These parameters should ensure that relations are established in the right way and lead to ethical decisions and actions. Table 2 summarizes and explains relational ethics features according to Pollard (18).

**Table 2:** Relational ethics features (18).

**Feature**	**Short description**
Mutual respect	Refers to the responsibility to the others (patients).
Engagement	To establish an engaged relationship with others (patient-provider relationship).
Embodied knowledge	Getting to know patients' needs, preferences, values to guide and orientate decision-making processes (partnership and patients' narrative).
Environment	Refers to the relationship between the person and the context of the social environment (taking into account patients' needs, values, family, community, history).
Uncertainty	The difficulty of undertaking a course of action or making decisions due to value-based demands.

As mentioned above, PCC seems not only to embody relational ethics but goes even beyond by assuming that persons are not only central to medical treatments, but they are at par with their providers, thereby strengthening even more the relationship aspect (7). This explains why PCC implies the paradigm shift from the notion of “patient-centered” to that of “person-centered.” The shift delineates that the patient is a person, a human being, with a unique background, relationships, capabilities, resources, strengths, and limitations.

Based on these assumptions, the aim of this scoping review is to explore whether or not PCC, in its quest to deliver high quality and safe health care, has a relational-ethics perspective. The research questions that guided this paper are: (i) to what extent is person-centered care related to relational ethics?; and (ii) what is the value of PCC as an ethical approach in delivering higher quality and safer healthcare?

The next sections of this paper will present: (i) the methodology and research strategy conducted for this review; (ii) results; and (iii) a discussion of results, main limitations of this study and further research directions.

## Methods

A scoping review was conducted to explore and understand the relation between PCC and relational ethics in the quest to deliver high quality and safe care in health care systems.

Scoping reviews are used to map concepts underpinning a research area and the main sources and types of evidence available. Scoping reviews are now seen as a valid approach in those circumstances where systematic reviews are unable to meet the necessary objectives or requirements of knowledge users. Due to lack of consistency existing in the terminology, definition, methods, and reporting of scoping reviews appearing in the literature, it is recommended to use PRISMA (Preferred Reporting Items for Systematic Reviews and Meta-Analyses) statement for the reporting scoping articles (32) (Table 3).

**Table 3:** Preferred Reporting Items for Systematic reviews and Meta-Analyses extension for Scoping Reviews (PRISMA-ScR) Checklist.

**Section**	**Item**	**PRISMA-ScR checklist item**	**Reported on page #**
**TITLE**			
Title	1	Identify the report as a scoping review.	1
**ABSTRACT**			
Structured summary	2	Provide a structured summary that includes (as applicable): background, objectives, eligibility criteria, sources of evidence, charting methods, results, and conclusions that relate to the review questions and objectives.	1
**INTRODUCTION**			
Rationale	3	Describe the rationale for the review in the context of what is already known. Explain why the review questions/objectives lend themselves to a scoping review approach.	1–4
Objectives	4	Provide an explicit statement of the questions and objectives being addressed with reference to their key elements (e.g., population or participants, concepts, and context) or other relevant key elements used to conceptualize the review questions and/or objectives.	4
**METHODS**			
Protocol and registration	5	Indicate whether a review protocol exists; state if and where it can be accessed (e.g., a Web address); and if available, provide registration information, including the registration number.	N/A
Eligibility criteria	6	Specify characteristics of the sources of evidence used as eligibility criteria (e.g., years considered, language, and publication status), and provide a rationale.	4–5
Information sources	7	Describe all information sources in the search (e.g., databases with dates of coverage and contact with authors to identify additional sources), as well as the date the most recent search was executed.	5
Search	8	Present the full electronic search strategy for at least 1 database, including any limits used, such that it could be repeated.	5
Selection of sources of evidence	9	State the process for selecting sources of evidence (i.e., screening and eligibility) included in the scoping review.	5
Data charting process	10	Describe the methods of charting data from the included sources of evidence (e.g., calibrated forms or forms that have been tested by the team before their use, and whether data charting was done independently or in duplicate) and any processes for obtaining and confirming data from investigators.	5
Data items	11	List and define all variables for which data were sought and any assumptions and simplifications made.	5
Critical appraisal of individual sources of evidence	12	If done, provide a rationale for conducting a critical appraisal of included sources of evidence; describe the methods used and how this information was used in any data synthesis (if appropriate).	N/A
Synthesis of results	13	Describe the methods of handling and summarizing the data that were charted.	N/A
**RESULTS**			
Selection of sources of evidence	14	Give numbers of sources of evidence screened, assessed for eligibility, and included in the review, with reasons for exclusions at each stage, ideally using a flow diagram.	6
Characteristics of sources of evidence	15	For each source of evidence, present characteristics for which data were charted and provide the citations.	6–8
Critical appraisal within sources of evidence	16	If done, present data on critical appraisal of included sources of evidence (see item 12).	N/A
Results of individual sources of evidence	17	For each included source of evidence, present the relevant data that were charted that relate to the review questions and objectives.	6–8
Synthesis of results	18	Summarize and/or present the charting results as they relate to the review questions and objectives.	6–8
**DISCUSSION**			
Summary of evidence	19	Summarize the main results (including an overview of concepts, themes, and types of evidence available), link to the review questions and objectives, and consider the relevance to key groups.	8–9
Limitations	20	Discuss the limitations of the scoping review process.	9
Conclusions	21	Provide a general interpretation of the results with respect to the review questions and objectives, as well as potential implications and/or next steps.	10
**FUNDING**			
Funding	22	Describe sources of funding for the included sources of evidence, as well as sources of funding for the scoping review. Describe the role of the funders of the scoping review.	11

*From: Tricco et al. (32)*.

Methodology of this review followed the steps as below: (i) inclusion and exclusion criteria for selecting articles were first identified; (ii) a search strategy was decided upon common agreement between all authors involved in this study; (iii) databases and keywords were identified according to the theoretical framework upon which this review was based; and (iv) division of tasks for reviewing articles and data extraction tools were agreed.

### Inclusion/Exclusion Criteria

Inclusion/exclusion criteria were established according to timespan, language, paper focus, research type, publication type, and research area. We searched for articles published from 1st January 2008 up to 30th November 2018 in order to include articles published in the last decade. We included only articles written in English, as it is the working language used by the authors involved in this study. Regarding papers' focus, we decided to include articles providing evidence on: (i) person-centered care; (ii) patient-centered care; and (iii) ethics/relational ethics person-centered care (or person-centered care). Papers not providing any of the aforementioned themes were excluded. Regarding research type, we included only papers based on primary search. We included in our databases search only papers/articles published in peer-reviewed journals, excluding website, documents, media articles, and other non-research documents since they do not offer research-based evidence. Furthermore, to avoid a large number of publications, we decided to exclude books and book chapters, dissertations, research reports, and conference proceedings. Finally, to avoid the inclusion of papers not pertinent to the aim of this paper, we considered only articles within the areas of: medicine; nursing; social sciences; psychology; health professions; biochemistry, genetics, and molecular biology; neuroscience; dentistry; pharmacology, toxicology and pharmaceutics. Although we have considered to include papers within other fields, notably ethics, we wanted to stick to the perspectives emanating only from those specialties that provided explanations on person-centered care, which is our main focus.

Regarding types of studies, qualitative, quantitative, as well as mixed studies, literature reviews and commentaries were included. Furthermore, no geographical limitations were applied in the search strategy.

Table 4 illustrates inclusion and exclusion criteria adopted in this work.

**Table 4:** Inclusion/exclusion criteria and main justification.

**Item**	**Inclusion criteria**	**Exclusion criteria**	**Main justification**
Timespan	All articles from 2008 onwards	Articles published before 2008	Papers published in the last decade.
Language	Papers written in English	Papers not written in English	English is the working language of the reviewers
Paper focus	Papers which provide evidence on: • Person-Centered care • Patient-Centered care • Ethics/relational ethics	Papers which do not provide evidence on person-centered care, patient-centered care, ethics/relational ethics.	This criterion is justified by the review questions
Research type	Papers based on primary research	Papers not based on primary research Opinion articles	This criterion is justified by the review questions
Publication type	Papers/articles published in peer-reviewed journals based on both theoretical and empirical research	Website documents, media articles and other non-research documents Books and book chapters, dissertations, research reports, conference proceedings	Website documents, media articles and other non-research documents were excluded because they do not offer research-based evidence Books and book chapters, dissertations, research reports, conference proceedings were excluded to avoid a large volution of publications
Research area	Papers within the areas of: medicine; nursing; social sciences; psychology; health professions; biochemistry, genetics and molecular biology; neuroscience; dentistry; pharmacology, toxicology and pharmaceutics;	Papers whose research area is different from the listed ones	To avoid the inclusion of papers not pertinent to aim of this work.

### Search Strategy

PCC and relational ethics were analyzed separately in order to identity their key features according to the literature as illustrated in Table 2. Upon reviewing the literature arising from the search, we elicited a framework for PCC and relational ethics features (Tables 1, 2).

This research was conducted using the following databases: Scopus, PubMed, and Medline. The choice of these databases was due to their consolidate reputation among research community.

The following combination of keywords was used to search in Scopus and Medline database search: “person-centered care” AND “mutual respect” OR engagement OR “embodied knowledge” OR environment OR uncertainty.

While searching articles in PubMed, since its software characteristics did not allow us to use the same strategy adopted in Scopus and Medline, we adopted the following combination of keywords: “person-centered care” AND “mutual respect”; “person-centered care” AND engagement; “person-centered care” AND “embodied knowledge”; “person-centered care” AND environment; “person-centered care” AND uncertainty.

### Selection of Publications

**Figure 1:**
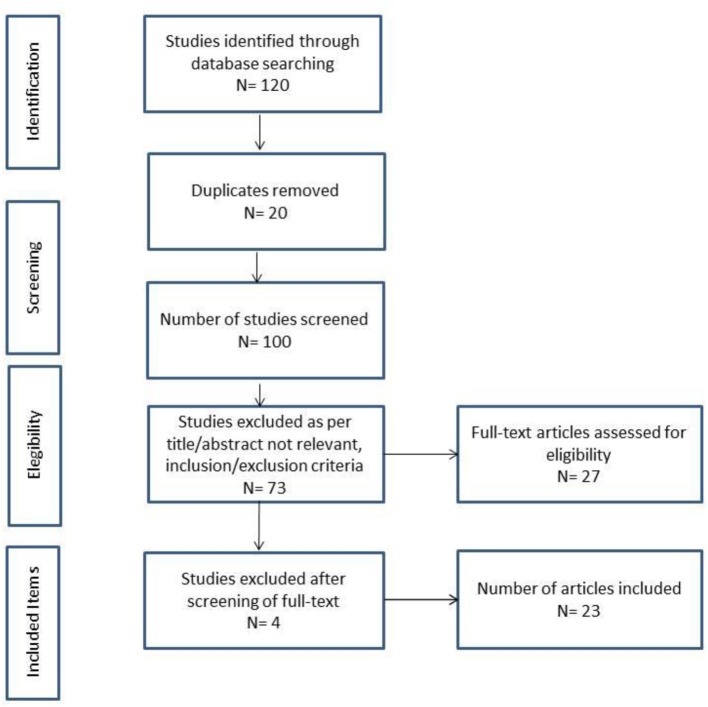
PRISMA flow chart.

The (PRISMA) flow diagram (33) illustrated in Figure 1 shows the number of publications that were selected in the different phases of this scoping review and the final total of articles included.

### Data Extraction and Synthesis

All articles were reviewed by all authors involved in this study, with an equal division of tasks. All relevant data contained in the reviewed articles were extracted to a data extraction form. The final version of the data extraction form includes the following items: article number; reference; year; author(s); title; country, health service; setting; aim(s)/objective(s); research design; method; population/sample; findings; and constructs variables. Each selected paper was reviewed in view of determining whether the identified construct features/actions were directly or indirectly mentioned.

Results are presented as below: (i) the general results (results of search screening, number of articles per year, country, and methodology approach) are first presented; (ii) the matches between PCC features and relational ethics actions identified in reviewed articles are further analyzed and discussed; and (iii) article groups are finally identified according to their communalities with regard to the framework considered in this scoping review.

## Results

Results of this scoping review allowed us to explore the relationships between PCC features and relational ethics actions. The analysis of the findings of the review exercise enabled us to understand the PCC -relational ethics perspective. The relationship between direct and indirect PCC features and relational ethics actions were identified.

### General Results

The searches in electronic databases yielded a total of 120 publications (*N* = 120). After removing 20 duplicates (*N* = 20), the total of published items to screen shifted to 100 (*N* = 100). After having applied the inclusion/exclusion criteria to titles and abstracts, 73 publications (*N* = 73) were excluded. Most of the screened publications were excluded because they were not pertinent to the research questions guiding this study and/or because they were not research papers. Upon the full-text screening, 4 publications (*N* = 4) were excluded since they were not pertinent to the research conducted in this paper. At the end of the process, 23 articles (*N* = 23) were included in this review (Figure 1). Article details can be retrieved from Supplementary Material attached to this review according to author(s), year, direct and indirect relational ethics framework, and direct and indirect PCC framework.

Most of the reviewed articles were published from 2015 onwards with a relatively peak of publications (*N* = 5) in 2016. Most of the included papers were published in Sweden (26%), United States of America and United Kingdom (22%). The studies which were screened included both qualitative and quantitative studies, as well as mixed method research studies, literature reviews and commentaries. The majority of papers were qualitative (31%) and quantitative studies (26%), followed by mixed method studies (17%). The minority of papers were literature reviews and commentaries (13%).

### PCC and Relational Ethics

**Figure 2:**
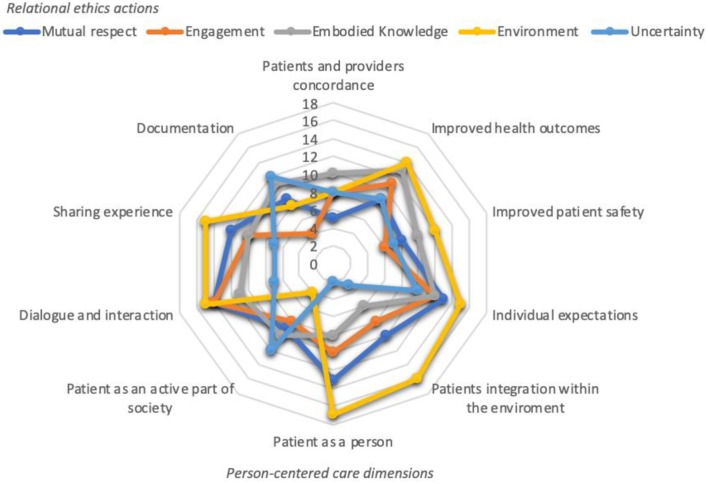
PCC and relational ethics framework.

Findings of this scoping review revealed matches and congruences between PCC and relational ethics. By doing so, we analyzed and compared PCC and relational ethics' dimensions that were mentioned (both directly and indirectly) in the reviewed papers (Figure 2).

As Figure 2 illustrates, there is congruence between PCC and relational ethics. The relationship between the person and the context of the social environment was the most recurring relational ethics action that was mentioned in reviewed papers together with the different PCC features. However, PCC emphasizes the role of the patient to that of a unique person, as a partner in care, as a co-creator of his/her healthcare plan, and integrated within the environment—social and other that the person is connected to. This does not seem to be clearly accentuated in defining relationships as part of relational ethics. Nevertheless, patient as a person, patients' integration within the environment, individual expectations, dialogue and interaction, and sharing experience followed by improved health outcomes, and improved patient safety are the most frequently PCC features mentioned in that order together with the relational ethics action environment.

Relationships were also observed with regard to relational ethics actions of embodied knowledge, mutual respect, and engagement and PCC features of improved health outcomes, improved patient safety, individual expectations, patient as an active part of society, dialogue and interaction, sharing experience, and documentation.

Uncertainty, namely the difficulty of undertaking a course of action or making decisions due to value-based demands, was the least stressed relational ethics action from Pollard's framework. It was sought to be related mostly to PCC features of improved health outcomes, individual expectations and documentation.

### Article Groups

While analyzing the results of the 23 articles, we identified three groups of articles (Table 5) according to the framework taken into considerations. Group 1 is the largest one with 17 articles, followed by group 3 (3 articles) and group 2 (2 articles). Only one article (56) was identified as not representing none of the three groups.

**Table 5:** Article groups.

**Group**	**References**	**PCC**	**Relational ethics**
1	Abbott et al. (34) Boscart et al. (35) Coyne et al. (36) Edvardsson et al. (37) Edvardsson et al. (38) Elfstrand Corlin et al. (39) Hung et al. (40) Alharbi et al. (41) Dudas et al. (42) Fawcett and Rhynas (43) Howard et al. (44) Johnston et al. (45) Kuluski et al. (46) Sjögren et al. (47) Stanhope et al. (48) McCormack et al. (49) Mills et al. (50)	Improved health outcomes Improved patient safety Individual expectations Dialogue and interaction Sharing experience Documentation Patients and providers concordance Patients integration within the environment Patient as a person Patient as an active part of his/her healthcare plan and of society	Mutual respect Embodied knowledge Environment Uncertainty Engagement
2	Geboy (51) Røsvik et al. (52)	Individual expectations Patients integration within the environment Dialogue and interaction Improved patient safety Patient as a person Patient as an active part of his/her healthcare plan and of society Sharing experience	Engagement Environment Uncertainty
3	Røen et al. (53) Rubashkin et al. (54) Scales et al. (55)	Patient as a person Individual expectations Patient as an active part of his/her healthcare plan and of society Documentation	Environment Embodied knowledge Uncertainty

As per Table 4, almost all PCC features and relational ethics actions were mentioned in articles belonging to group 1 (34–50).

In group 2 (51, 52), individual expectations, patients' integration within the environment, dialogue and interaction, improved patient safety, patient as a person, patient as an active part of society and sharing experience were the most recurring PCC features mentioned together with relational ethics actions of engagement, environment and uncertainty.

In group 3 (53–55), PCC features of patient as a person, individual expectations, patient as an active part of society and documentation were mentioned in conjunction with relational ethics actions of environment, embodied knowledge and uncertainty.

Finally, in English (56)—which was not belonging to none of the aforementioned group—patients and providers' concordance, improved health outcomes, individual expectations, patient as a person, dialogue and interaction and sharing experience were mentioned together with relational ethics actions of mutual respect, engagement, uncertainty, and embodied knowledge.

## Discussion

### Summary of Main Findings

Results emerging from the 23 articles included in this scoping review suggest that there is a notable relationship between PCC features and relational ethics actions. However, PCC seems to go beyond and raises the provider-patient relationship to a higher ethical approach, namely that partner in care and co-creator of the healthcare plan.

Nevertheless, the relevance of embodied knowledge, mutual respect, engagement and uncertainty within the caring environment was identified as the most recurrent relational ethical dimension in relation to the majority of PCC features.

Thus, there is certainly a match between PCC features and relational ethics dimensions as discussed in Pollard's framework. If we look at the three pillars of PCC—partnership, patients' narrative, and documentation—we can sum up that the articles reviewed in this scoping review addressed these areas in almost their entirety, although only exclusively addressed by Ekman and her colleagues. The relationship between the two concepts stands in the assumption that while health professionals are establishing a partnership with their patients and listening to their stories, they are exercising an ethical behavior at the same time.

Furthermore, we can summarize communalities between PCC features and relational ethics actions. In this respect, the PCC feature of patient as a person is totally embraced by the relational ethics action of mutual respect. Treating patients as persons finds its ethical foundations in the mutual respect concept, which is referred to physicians' social and ethical responsibilities toward their patients. PCC pillars of partnership and patients' narrative can be connected to relational ethics actions of embodied knowledge and engagement. Getting to know patients' needs, preferences, values, as well as establishing an engaged relationship between the patient and the health provider are key enablers that orientate decision-making processes.

Moreover, the combination of the above-mentioned concepts may help health providers to better know their patients with a consequent reduction of uncertainty (a further relational ethics action which was less stressed in the reviewed articles). As difficulties of understanding specific courses of action or decision making (due to value-based demands) may arise, patients' narrative—by involving a deeper knowledge and an effective engagement between patients and providers—may help lowering the levels of uncertainty. Finally, relational ethics action of environment matches with PCC features of patients' integration within the environment and patient as part of society. In this regard, the relationship between the patient (person) and the social context (family, friends, community, patients' history) assumes an important role in orientating treatment plans.

This work has implications for both theory, research, and practice. Regarding theoretical and research implications, this study provides a contribution to on-going academic debate on the ethical foundations of person-centered approaches to health care. As regards practical implications, by relating PCC and relational ethics approaches within health organizations—one can appreciate the differences and hence the higher ethical level contribution of PCC in not only establishing ethical provider-patient relationships but in elevating this relationship on the level of partnership and co-creation of the healthcare plan, and therefore both having active roles in clinical decision-making while fully embracing the biopsychosocial model of care. Furthermore, PCC considers patients as assets within the health system by appreciating their capabilities and resources. The most notable practical implication is if PCC is formalized into policy documents. This may be a driver for reaching higher quality standards and safer health care. From a policy-making point of view, formalizing these actions into official documents would allow a better implementation of PCC and relational ethics within healthcare organizations' strategies. By considering patients as persons-partners, organizations are contributing to patients' empowerment and are recognizing their role as active part in care and of society.

Treating patients as persons, partners, mutual respect, embodied knowledge, and engagement—in order to enhance quality and safety of care—are the key points emerging from this research.

However, these principles are not new to medicine and have their foundations back to Aristotle and Hippocrates times (400 BCE). Hippocrates' focus was not just on the mere treatment of patients' diseases but also on the individual as a person. To this extent, his school set up a code of conduct (moral and personal code of conduct) identifying persons' needs as the pillars of care (57). In the same line, Galen's philosophy (centuries later) put the individual first as well as William Osler (between the nineteenth and twentieth century), whose focus was on the individual as a person. In this regard, Osler's contribution was fundamental for the further development of PCC philosophy. His quotation “listen to your patient, he is telling you the diagnosis” already incorporated PCC's principles of establishing a patient-provider relationship and emphasized the importance of listening and documenting patients' needs (58).

### Strengths and Limitations

There are both strengths and limitations in the scoping review conducted for this paper. We identified subjectivity of reviewers as one of the main limitations of this study. This is common a weakness of qualitative analysis. Lack of language pluralism in publications selected by the research team is another limitation of this work, as only primarily publications in English language were included (articles written in any other language were excluded). Furthermore, books, book chapters and conference proceedings were excluded, and this may have further compromised the number of published items which were screened. A further limitation lies in the use of a limited set of databases (namely, Scopus, Medline, and PubMed) for our search strategy and thus some relevant papers which were not listed on the referred three databases may have been missed. However, having included both qualitative and quantitative studies, as well as mixed studies, literature reviews and commentaries—without geographical limitations—rendered strength to this review.

This scoping review took a broad approach with regard to geographical areas and did not take into consideration differences between countries when it comes to the different types of political and health systems. In this respect, the way that PCC is implemented in publicly funded health systems may be different compared to how it is applied within market-oriented systems. Thus, further research is needed to reach a higher level of understanding of PCC from an ethical perspective and its application within different health systems.

## Conclusions

The aim of this scoping review is to explore whether or not person-centered care (PCC), in its quest to deliver high quality and safe health care, has a relational-ethics perspective. The results of the review suggested that PCC dimensions are closely linked to relational ethics actions and therefore confirms Ekman et al. (7) assertion that person-centered care is an ethical philosophy of care. Indeed, due to its characteristics, PCC already incorporates relational ethics within itself. As noted earlier, PCC draws upon the contention that the patient is regarded as a person with one's own unique characteristics, background, history, strengths and limitations. The person is not only a focal point in a health system as is described in patient-centered care but as a partner and co-creator of the healthcare plan as is emphasized in PCC. In person-centered care, the patient shifts from a passive role to an active role and utilizes his/her capabilities and resources. The foundations of this approach can be retrieved back to Hippocrates time and, more recently, Osler who emphasized the importance of patient-provider relationship and listening to patients' history in order to identify most suitable treatment plans. This strong relationship between patients and health providers is required not only to agree on treatments, services and care delivery but also to incorporate and document the person's needs (health, social, psychological, work, family, society), expectations and wishes. In so doing, it is envisaged that quality of care will be higher, and costs will be better contained. All is to be based on patients' characteristics, needs, and expectations (7).

However, while reviewed articles showed that PCC features and pillars are embraced by relational ethics actions, the way that PCC is practiced seems to be lacking for some authors, and for other authors PCC seems to be closer to the relational ethics framework. Thus, PCC may not be understood or practiced in its entirety within the relational ethical framework, so much so that Rockwell (59) critiqued PCC in residential care facilities as remaining within the biomedical model and concluded and recommended to expand the focus to relational care. This means that not always PCC encompasses entirely the relational ethics components. This observation also emerged from reviewed articles, where for example the relational ethics action of uncertainty was not recurrent and the other actions.

Furthermore, PCC goes beyond the relational ethics framework in the way that features such as documentation and patients' narrative enhance the way of communication between patients and providers. In this respect, communication is not just verbal and/or visual, but it is also formalized into official documents being thus part of management plans and decision-making processes. In a PCC approach, the persons as patients have capabilities and resources that the PCC relationship should uncover to ensure provider-patient partnership and the emphasis on the patient's narrative that can be documented. PCC relationship leads to the provider-patient co-creation of the healthcare plan.

In conclusion, this paper reveals that while PCC features embrace most of the relational ethics approaches, these are not exploited in their entirety and therefore PCC emerges as a unique ethical stance in healthcare. PCC's ethical approach goes beyond what is explained within provider-patient relational ethics and emphasizes that the patient as an active person with capabilities and resources. This distinction enables us to explain the paradigm shift from “patient-centered” to “person-centered” care. The healthcare provider partnership and co-creation of the healthcare plan contributes to the delivery of high quality, safe and cost-contained healthcare.

## Author Contributions

GT and SB conceived the idea, developed the methodology/search strategy, and lead the manuscript. Furthermore, they were involved in writing, editing, and reviewing the manuscript. AR and MC contributed in the review of articles and editing of the paper. GG contributed in the first development of the idea/concept and in the final editing the manuscript.

### Conflict of Interest

The authors declare that the research was conducted in the absence of any commercial or financial relationships that could be construed as a potential conflict of interest.
