# Supplementary material for: Person-Centered Care From a Relational Ethics Perspective for the Delivery of High Quality and Safe Healthcare: A Scoping Review
Source: Front Public Health. 2020 Mar 6;8:44. doi: 10.3389/fpubh.2020.00044 (PMC7067745; doi:10.3389/fpubh.2020.00044)
Supplement: Supplementary file 1 [file Table_1.DOCX]

*Supplementary material – List of included articles*

| Author(s) / Year | Ethics explicit | PCC explicit | Ethics implicit | PCC implicit |
| --- | --- | --- | --- | --- |
| Abbott et al. (2016) | Mutual respect, Embodied knowledge, Environment, Uncertainty | Improved health outcomes, Improved patient safety, Individual expectations, Dialogue and interaction, Sharing experience, Documentation | Engagement | Patients and providers concordance, Patients integration within the environment, Patient as a person, Patient as an active part of society |
| Alharbi et al. (2012) | Embodied knowledge, Environment, Uncertainty | Patients and providers concordance, Improved health outcomes, Improved patient safety, Patients integration within the environment, Patient as an active part of society, Documentation | Mutual respect, Engagement | Individual expectations, Patient as a person, Dialogue and interaction, Sharing experience |
| Boscart et al. (2018) | Mutual respect, Engagement, Embodied knowledge, Environment | Improved health outcomes, Improved patient safety, Individual expectations, Dialogue and interaction, Sharing experience |  | Patients and providers concordance, Patients integration within the environment, Patient as a person, Patient as an active part of society |
| Coyne et al. (2018) | Mutual respect, Engagement, Embodied knowledge, Environment | Improved health outcomes, Improved patient safety, Individual expectations, Patient as a person, Dialogue and interaction, Sharing experience, Documentation |  | Patients and providers concordance, Patients integration within the environment, Patient as an active part of society, |
| Dudas et al. (2013) | Embodied knowledge, Uncertainty | Patients and providers concordance, Improved health outcomes, Individual expectations, Patient as a person, Sharing experience, Documentation | Mutual respect, Engagement, | Patients integration within the environment, Patient as an active part of society, Dialogue and interaction |
| Edvardsson et al. (2010) | Mutual respect, Embodied knowledge, Environment | Individual expectations, Patients integration within the environment, Patient as a person, Patient as an active part of society | Engagement | Patients and providers concordance, Improved health outcomes, Dialogue and interaction, Sharing experience |
| Edvardsson et al. (2014) | Mutual respect, Embodied knowledge, Environment | Patients and providers concordance, Improved health outcomes, Improved patient safety, Patients integration within the environment, Patient as a person, Dialogue and interaction, Sharing experience | Engagement, Uncertainty | Individual expectations, Patient as an active part of society, documentation |
| Elfstrand Corlin et al. (2017) | Mutual respect, Embodied knowledge, Environment, Engagement | Patients and providers concordance, Improved health outcomes, Individual expectations, Patient as a person, Dialogue and interaction, Sharing experience |  | Improved patient safety, |
| English (2016) | Mutual respect, Engagement, Uncertainty | Patients and providers concordance, Improved health outcomes, Individual expectations, Patient as a person, Dialogue and interaction, Sharing experience | Embodied knowledge |  |
| Fawcett & Rhynas (2014) | Embodied knowledge, Environment, Uncertainty | Patients and providers concordance, Improved health outcomes, Improved patient safety, Individual expectations, Patients integration within the environment, Patient as a person, Dialogue and interaction, Sharing experience, documentation | Mutual respect, Engagement, | Patient as an active part of society |
| Geboy (2009) | Engagement, Environment | Individual expectations, Patients integration within the environment, Dialogue and interaction | Uncertainty | Improved patient safety, Patient as a person, Patient as an active part of society, Sharing experience |
| Howard et al. (2016) | Embodied knowledge, Environment, Uncertainty, Engagement | Patients and providers concordance, Improved health outcomes, Individual expectations, Patients integration within the environment, Patient as a person, Dialogue and interaction, Sharing experience | Mutual respect | Improved patient safety, Patient as an active part of society |
| Hung et al. (2016) | Mutual respect, Embodied knowledge, Environment, Engagement | Patients and providers concordance, Improved health outcomes, Individual expectations, Patients integration within the environment, Improved patient safety, Patient as a person, Dialogue and interaction, Sharing experience | Uncertainty | Patient as an active part of society, documentation |
| Johnston et al. (2015) | Mutual respect, Environment | Individual expectations, Patients integration within the environment, Patient as a person, Dialogue and interaction, Sharing experience, documentation | embodied knowledge, Engagement, uncertainty | Patients and providers concordance, Patient as an active part of society |
| Kuluski et al. (2016) | Mutual respect, Environment | Individual expectations, Patients integration within the environment, Patient as a person, Dialogue and interaction, Patient as an active part of society | Embodied knowledge, Engagement, Uncertainty | Improved health outcomes, Improved patient safety, documentation |
| McCormack et al. (2011) | Mutual respect, Environment, Engagement | Patients integration within the environment, Patient as a person, Sharing experience, documentation | Embodied knowledge, Uncertainty | Patients and providers concordance, Improved health outcomes, Individual expectations, Improved patient safety, Patient as an active part of society, Dialogue and interaction |
| Mills et al. (2015) | Mutual respect, Environment, Engagement | Improved health outcomes, Individual expectations, Improved patient safety, Patients integration within the environment, Patient as a person, Dialogue and interaction | Embodied knowledge, Uncertainty | Patient as an active part of society, Sharing experience, documentation |
| Røen et al. (2018) | Environment | Patient as a person | Embodied knowledge, Uncertainty | Individual expectations, Patient as an active part of society, documentation |
| Røsvik et al. (2011) | Environment, Engagement | Improved health outcomes, Individual expectations, Improved patient safety, Patients integration within the environment, Patient as a person, Dialogue and interaction, Sharing experience | Mutual respect, Embodied knowledge, Uncertainty | Patient as an active part of society, documentation |
| Rubashkin et al. (2018) | Environment | Improved health outcomes, Improved patient safety, Patients integration within the environment, Patient as a person, Dialogue and interaction, Sharing experience | Mutual respect, Embodied knowledge, Uncertainty, Engagement | Individual expectations, Patient as an active part of society, documentation |
| Scales et al. (2017) | Environment | Patients and providers concordance, Improved health outcomes, Individual expectations, Patients integration within the environment, Patient as a person, Improved patient safety, Sharing experience | Mutual respect, Embodied knowledge, Uncertainty, Engagement | Patient as an active part of society, documentation, Dialogue and interaction |
| Sjögren et al. (2017) | Mutual respect, Environment | Patients and providers concordance, Improved health outcomes, Individual expectations, Patients integration within the environment, Patient as a person, Improved patient safety, Sharing experience, Patient as an active part of society, documentation, Dialogue and interaction | Embodied knowledge, Uncertainty, Engagement |  |
| Stanhope et al. (2015) | Mutual respect, environment | Patients integration within the environment, Patient as a person, Sharing experience, documentation | Engagement, Uncertainty, Embodied knowledge | Patients and providers concordance, Improved health outcomes, Individual expectations, Improved patient safety, Patient as an active part of society, Dialogue and interaction |
